# Screening Biomarker as an Alternative to Endoscopy for the Detection of Early Gastric Cancer: The Combination of Serum Trefoil Factor Family 3 and Pepsinogen

**DOI:** 10.1155/2018/1024074

**Published:** 2018-05-09

**Authors:** Hyun Seok Lee, Seong Woo Jeon, Sachiyo Nomura, Yasuyuki Seto, Yong Hwan Kwon, Su Youn Nam, Yuko Ishibashi, Hiroshi Ohtsu, Yasukazu Ohmoto, Hae Min Yang

**Affiliations:** ^1^Department of Internal Medicine, School of Medicine, Kyungpook National University, Kyungpook National University Hospital, Daegu, Republic of Korea; ^2^Department of Gastrointestinal Surgery, Graduate School of Medicine, University of Tokyo, Tokyo, Japan; ^3^Department of Breast and Endocrine Surgery, Graduate School of Medicine, University of Tokyo, Tokyo, Japan; ^4^Department of Clinical Trial Data Management, Graduate School of Medicine, University of Tokyo, Tokyo, Japan; ^5^Otsuka Pharmaceutical Co. Ltd., Tokyo, Japan

## Abstract

**Objective:**

The serum pepsinogen test has limitation in its predictive power as a noninvasive biomarker for gastric cancer screening. We aimed to investigate whether the combination of TFF3 and pepsinogen could be an effective biomarker for the detection of gastric cancer even in the early stages.

**Methods:**

In total, 281 patients with early gastric cancer (EGC), who underwent endoscopic submucosal dissection in Korea, and 708 healthy individuals from Japan were enrolled in the derivation cohort. The validation cohort included 30 Korean patients with EGC and 30 Korean healthy control blood donors. Serum TFF3 levels were examined using enzyme-linked immunosorbent assay.

**Results:**

Using a cutoff of 6.73 ng/mL in the derivation cohort, the sensitivity of the combination of tests for EGC detection was superior (87.5%) to that of TFF3 (80.4%) or pepsinogen test alone (39.5%). Similarly, in the validation cohort, the sensitivity of TFF3 plus pepsinogen was higher (90.4%) than that of TFF3 (80.0%) or pepsinogen test alone (33.3%).

**Conclusion:**

The combination of serum TFF3 and pepsinogen is a more effective noninvasive biomarker for gastric cancer detection compared with pepsinogen or TFF3 alone, even in EGC. This trial is registered with NCT03046745.

## 1. Introduction

Gastric cancer is the third leading cause of cancer death in the world [1–3]. Approximately half of the gastric cancer cases are diagnosed during advanced stages. One of the reasons for this is the invasiveness of esophagogastroduodenoscopy (EGD) screening examinations that leads to patients avoiding necessary tests [4]. The limitation of the pepsinogen test as a noninvasive serologic biomarker screening method is that the optimal cutoff value could be affected by several factors, such as *H. pylori* infection, age, gender, and the test method itself [5].

The trefoil factor family (TFF) of peptides comprises small (12–22 kDa) molecules that are secreted by the mammalian gastrointestinal tract. They are extremely stable in acidic conditions and resistant to heat degradation and proteolytic digestion. TFFs constitute a family of three peptides (TFF1, TFF2, and TFF3) that are widely expressed in a tissue-specific manner in the gastrointestinal tract. TFF3 is expressed in the goblet cells of the small and large intestines as well as the intestinal metaplasia in the stomach [6–10]. Serum TFF3 was shown to be a better potential screening tool for gastric cancer than pepsinogen in Japan [11].

These characteristics of TFF3 prompted us to analyze whether serum TFF3 can be a biomarker of early gastric cancer (EGC) in Koreans, as well as in the Japanese population. There is no previous study on serum TFF3 as a biomarker in EGC population without advanced gastric cancer (AGC). Because the detection of early-stage cancer is associated with improved survival, our hypothesis is that the combination of TFF3 with pepsinogen could enhance the sensitivity of EGC detection. Thus, we investigated if the combination of serum TFF3 and pepsinogen tests could be a more effective noninvasive tool for the detection of EGC.

## 2. Materials and Methods

### 2.1. Subjects

#### 2.1.1. Study Population of Derivation Cohort

The patient group consisted of 281 EGC patients who underwent endoscopic submucosal dissection at the Kyungpook National University Medical Center in Korea from January 2011 to May 2013. We obtained blood samples from all the patients before their endoscopic treatment. The control group consisted of 708 healthy male and female blood donors who had received a health check at Yamanaka Clinic in Japan from October 2011 to December 2012. The biopsy specimens for this study were provided by the National Biobank of Korea, Kyungpook National University Hospital (KNUH), which is supported by the Ministry of Health, Welfare and Affairs. All materials derived from the National Biobank of Korea, KNUH, were obtained under institutional review board-approved protocols.

#### 2.1.2. TFF3 Value in the Validation Cohort

In the derivation cohort of the current study, the control group consisted of Japanese individuals, and we have not obtained the results of serum pepsinogen in the control group. In order to test our results in another validation cohort with both patients and controls from the same country, the Korean validation cohort was needed. The validation cohort was an independent cohort, containing 30 Korean EGC patients from August 2016 to December 2016 and 30 Korean healthy control blood donors who received a health check including EGD from August 2016 to December 2016. Their data were prospectively collected and analyzed to validate the TFF3 value. The study protocols used for subjects in the validation cohort were identical to those used for subjects in the derivation cohort. The validity of the combination of TFF3 and pepsinogen for the detection of EGC in Korean individuals was assessed by receiver operating characteristic (ROC) analysis.

### 2.2. Methods

#### 2.2.1. Construction of Human TFF3 Expression Plasmids

Human TFF3 complementary deoxyribonucleic acid (cDNA) was cloned from Human Small Intestine Marathon-Ready cDNA (Clontech, Mountain View, CA, USA) by polymerase chain reaction. For His-tagged *Escherichia coli* expression, the human TFF3 cDNA fragments were inserted into the pET-21a(+) (Novagen) vector to create pET-hTFF3-His [11].

#### 2.2.2. Expression and Purification of Recombinant Human TFF3

BL21-CodonPlus (DE3)-RIL bacteria (Stratagene, Santa Clara, CA, USA) were transformed with the pET-hTFF3-His plasmid and then cultured in lysogeny broth medium at 37°C. Recombinant protein expression was induced by incubating cells with 1 mmol/L isopropyl *β*-D-1-thiogalactopyranoside for 5 hours. Bacterial pellets were harvested, the soluble protein fractions were extracted by sonication in 0.2% Triton X-100 and 50 mmol/L Tris-HCl (pH 8.0), and recombinant human TFFs were purified by Ni-Resin chromatography (Invitrogen, Tokyo, Japan). Recombinant human TFFs were eluted from the Ni-Resin column with 0.5 mol/L imidazole, 50 mmol/L Tris-HCl (pH 8.0), and 0.5 mol/L NaCl. Each elution fraction was analyzed by performing sodium dodecyl sulfate polyacrylamide gel electrophoresis and Western blot analysis. Concentrations of the purified recombinant human TFFs were measured by using a protein assay (Bio-Rad Laboratories Inc., Tokyo, Japan) [11].

#### 2.2.3. Immunoassays for TFF3, Pepsinogen I, Pepsinogen II, and *Helicobacter pylori* Infection Status

Serum TFF3 levels were measured by performing enzyme-linked immunosorbent assay (ELISA). Antisera were prepared from rabbits immunized with human TFFs. The sensitivity of TFF3 was 30 pg/mL. Serum pepsinogen I and pepsinogen II levels were measured using the latex-enhanced turbidimetric immunoassay (Hitachi Ltd, Tokyo, Japan), and pepsinogen I/pepsinogen II ratio was calculated.

A positive *H. pylori* infection status was dependent on at least one of the following tests showing evidence of infection: histology, rapid urease test, and [^13^C]-urea breath test.

### 2.3. Statistical Analysis

All statistical analyses were performed using JMP7 software (SAS Institute Inc., Cary, NC, USA) or SPSS version 14.0 (SPSS Inc., Chicago, IL, USA). The mean of variables was compared between two groups using a *t*-test. The ROC curve for each evaluation was used to extract the corresponding cutoff point, which can be used to discriminate different gastric statuses. For that purpose, the area under each ROC curve was used to measure the discriminatory ability of the model. The resulting value of the cutoff point for each evaluation was applied to the determination of the sensitivity, specificity, and odds ratio. Consequently, 95% confidence intervals were calculated. A 2-sided *P* value of less than 0.05 was considered statistically significant.

## 3. Results

### 3.1. Characteristics of the Derivation and Validation Cohorts

**Figure 1:**
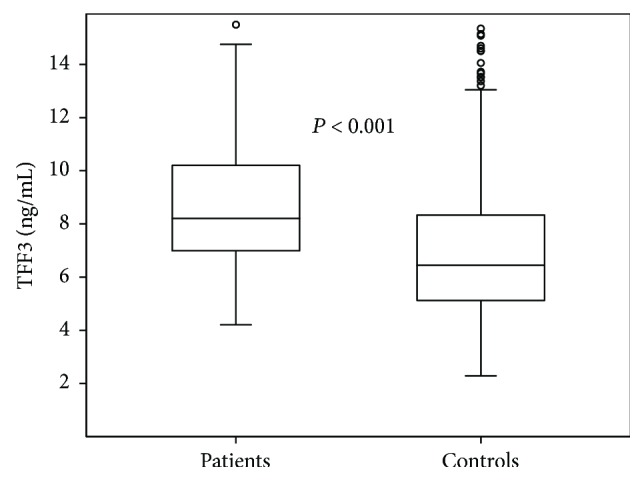
Serum trefoil factor family 3 (TFF3) levels in patients with gastric cancer were compared with healthy control individuals in the derivation cohort. The TFF3 level was significantly higher in patients with gastric cancer (*P* < 0.001).

In the derivation cohort, there were 217 (75.8%) male patients in the EGC group and that of the control group were 272 (38.4%). The mean age of patients in the cancer group was 63.4 ± 9.3 years, and that of the controls was 67.4 ± 11.9 years. The rate of positive *H. pylori* infection in the cancer group was 48.4%. Of the 281 studied tumors, 256 (91.1%) were histologically classified as differentiated type and 25 (8.9%) as undifferentiated type (Table 1). The mean serum TFF3 level in the patients with gastric cancer was 9.37 ± 4.67 ng/mL, which was significantly higher compared with that in the control group (7.05 ± 3.28 ng/mL; *P* < 0.001; Table 1, Figure 1).

For the validation cohort, 30 Korean EGC patients and 30 Korean healthy control subjects were enrolled (Table 1). There were 21 (70.0%) male patients in the EGC group and 15 (50.0%) in the control group. The mean age of EGC patients was 59.5 ± 10.7 years, and that of the controls was 66.6 ± 12.0 years. The mean serum TFF3 level in patients with gastric cancer was 9.01 ± 4.21 ng/mL, which was significantly higher than that in the control group (6.92 ± 2.76 ng/mL; *P* < 0.001).

### 3.2. Effect of *H. pylori* Infection on Serum TFF3 Levels in the Derivation Cohort

To test the diagnostic accuracy of serum TFF3 for identifying *H. pylori* infection among patients with cancer, ROC analysis was performed (data not shown). The area under the ROC curve of TFF3 was 0.445.

**Figure 2:**
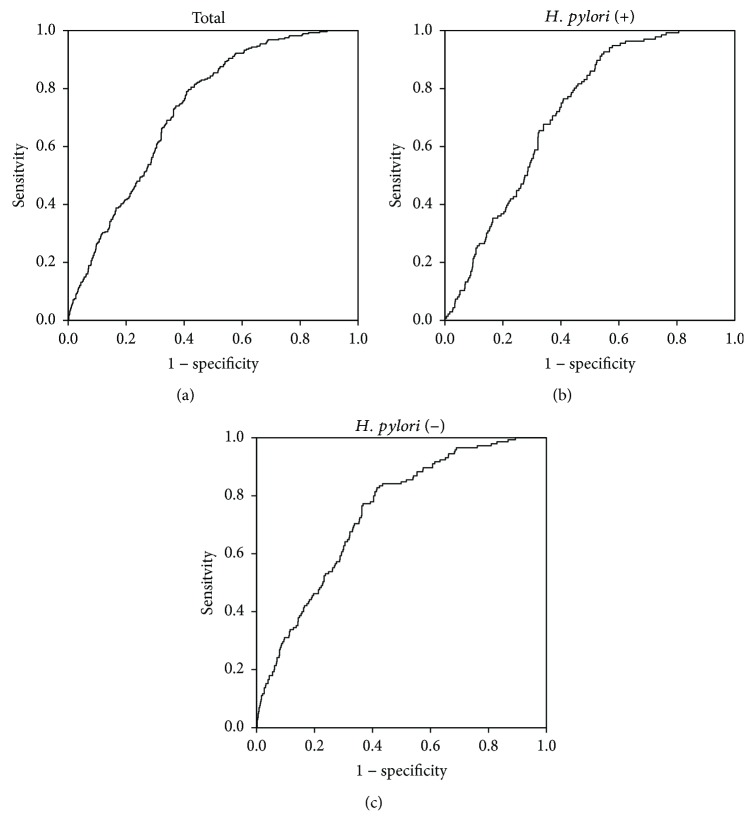
Receiver operating characteristic (ROC) curves of trefoil factor family 3 (TFF3) to predict early gastric cancer presence in the derivation cohort. (a) ROC curve of serum TFF3 for all (both *Helicobacter pylori*-positive and *Helicobacter pylori*-negative) the patients. The sensitivity, specificity, odds ratio, area under the curve, and cutoff value of TFF3 were 0.804, 0.576, 5.60, 0.729, and 6.73, respectively. The positive and negative predictive values of TFF3 were 0.430 and 0.881, respectively. (b) For *H. pylori*-positive patients, the sensitivity, specificity, odds ratio, and area under the curve were 0.772, 0.576, 4.61, and 0.716, respectively. (c) For *H. pylori*-negative patients, the sensitivity, specificity, odds ratio, and area under the curve were 0.835, 0.576, 6.86, and 0.740, respectively.

To test the diagnostic accuracy of serum TFF3 for identifying EGC, ROC analysis was performed. For both *H. pylori*-positive and *Helicobacter pylori*-negative patients, the sensitivity, specificity, odds ratio, area under the curve, and cutoff value for TFF3 were 0.804, 0.576, 5.60, 0.729, and 6.73, respectively. The positive and negative predictive values for TFF3 were 0.430 and 0.881, respectively (Figure 2(a)). To further evaluate TFF3, patients were divided according to *H. pylori* infection status and then ROC analysis was performed. The area under the curve was 0.716 for *H. pylori*-positive patients (Figure 2(b)) and 0.740 for *H. pylori*-negative patients (Figure 2(c)).

### 3.3. Histologic Types and Serum TFF3 Levels in the Derivation Cohort

**Figure 3:**
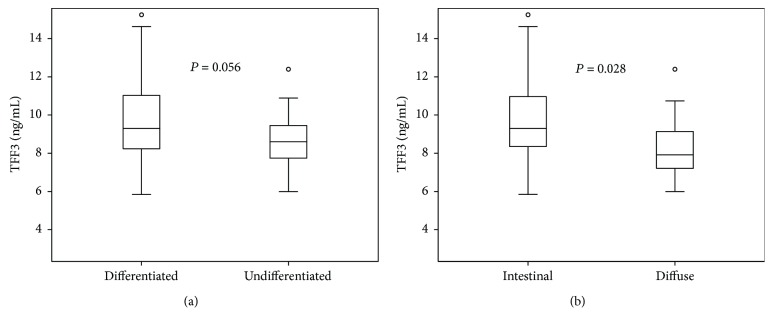
Distribution of serum trefoil factor family 3 (TFF3) in differentiated or undifferentiated-type and intestinal- or diffuse-type early gastric cancer (EGC) in the derivation cohort. (a) The serum TFF3 levels of patients with differentiated-type histology and of those with undifferentiated-type EGC did not differ significantly (*P* = 0.056). (b) Serum TFF3 levels in patients with intestinal-type EGC was significantly higher than that in those with diffuse-type cancer (*P* = 0.028).

To test the influence of EGC on serum TFF3 levels, the TFF3 level in each patient's serum was compared with their EGC histologic types. Differentiated gastric cancer included cases with well-differentiated or moderately differentiated adenocarcinomas. Gastric cancer with undifferentiated-type histology included cases with poorly differentiated adenocarcinoma or signet ring cell carcinoma. Serum TFF3 levels of patients with the differentiated type and of those with the undifferentiated type of EGC did not differ significantly (9.53 ± 4.83 ng/mL versus 7.66 ± 1.82 ng/mL, respectively; *P* = 0.056). On the other hand, serum TFF3 level in patients with the intestinal-type EGC was significantly higher than in patients with the diffuse type (9.54 ± 4.78 ng/mL versus 7.16 ± 1.89 ng/mL, respectively; *P* = 0.028; Figure 3). In any other pathologic status of EGC, such as submucosal invasion or lymphovascular invasion, there was no significant difference in serum TFF3 levels (data not shown).

### 3.4. Combination of the Serum TFF3 and Pepsinogen Tests in the Derivation Cohort

We analyzed the usefulness of determining the TFF3 level together with pepsinogen testing. The number of patients with gastric cancer and positive or negative results for both tests in the present study is shown in Table 2. The cutoff values for defining a positive pepsinogen test were a serum pepsinogen I level of <70 ng/mL and serum pepsinogen I/II ratio of <3. Under these cutoff values, 170 of the 281 patients with EGC were shown not to have cancer with pepsinogen screening alone. However, when serum TFF3 testing was added to the gastric cancer screening, 135 of the 170 EGC patients who were not identified by pepsinogen testing could be identified by the TFF3 examination. On the other hand, 20 of the 281 patients were not detected by TFF3 testing but were detected by pepsinogen testing.

The sensitivity of the individual pepsinogen and TFF3 tests was 39.5% and 80.4%, respectively. With combination testing, the sensitivity for gastric cancer presence was 87.5%, which was higher than that of TFF3 testing alone.

### 3.5. Combination of the Serum TFF3 and Pepsinogen Tests in the Korean Validation Cohort

**Figure 4:**
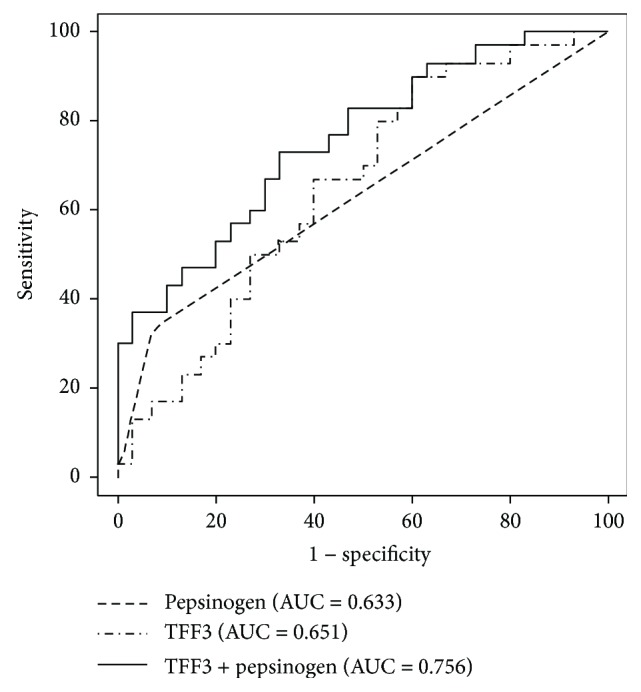
The Receiver operating characteristic curves of pepsinogen I/II ratio, serum trefoil factor family 3 (TFF3), and TFF3 plus pepsinogen I/II ratio are shown in the validation cohort. The sensitivity, specificity, odds ratio, and area under the curve of pepsinogen test for detection of EGC according to the definition of pepsinogen test were 0.333, 0.933, 7.00, and 0.633, respectively. Using the cutoff value of 6.73 ng/mL for TFF3, those for TFF3 were 0.800, 0.433, 3.06, and 0.651, respectively. Those for the combination of TFF3 and pepsinogen l/ll ratio were 0.900, 0.367, 5.21, and 0.756, respectively. AUC: area under the curve.

To test the diagnostic performance of pepsinogen test and serum TFF3 for identifying EGC in the Korean validation cohort, ROC analysis was performed (Figure 4). The sensitivity, specificity, odds ratio, and area under the curve of pepsinogen test for the detection of EGC according to the definition of pepsinogen test were 0.333, 0.933, 7.00, and 0.633, respectively. Using the cutoff value of 6.73 ng/mL for TFF3, those for TFF3 were 0.800, 0.433, 3.06, and 0.651, respectively. Those for the combination of TFF3 and pepsinogen l/ll ratio were 0.900, 0.367, 5.21, and 0.756, respectively.

The positive and negative predictive values of pepsinogen l/ll ratio were 0.833 and 0.583, respectively, and those of TFF3 were 0.585 and 0.684, respectively. Those of the combination of TFF3 and pepsinogen l/ll ratio were 0.587 and 0.786, respectively.

## 4. Discussion

The pepsinogen test is used for gastric cancer screening in Japan [4], where test sensitivity in population-based studies ranges from 71% to 84%, and specificity ranges from 57% to 78% [12]. In the present study, we compared serum TFF3 with serum pepsinogen test as serologic screening tools for detection of EGC in Korean patients. Sensitivity of the pepsinogen test was 39.5% in the derivation cohort, showing a lower sensitivity than those of the Japanese studies. It seems that the pepsinogen test for gastric cancer is easily influenced by various factors, including *H. pylori* status and the test method itself, and therefore does not meet the ideal screening criteria [5, 13, 14]. On the other hand, the serum TFF3 test showed higher sensitivity (80.4%) than pepsinogen test for detecting EGC in our study. Moreover, results of the serum TFF3 test were not influenced by *H. pylori* status. Similarly, recent Japanese study on 1260 healthy individuals showed that serum TFF3 values were not considerably affected by *H. pylori* status and eradication [15]. The authors suggested that serum TFF3 could be a stable biomarker of gastric cancer even after *H. pylori* eradication in contrast with the pepsinogen test [15]. Because TFF3 is not expressed in epithelial cells of the stomach and is only expressed in the intestinal goblet cells of the metaplasia, serum TFF3 levels are less influenced by *H. pylori* infection [15–17].

The TFFs that consist of TFF1, TFF2, and TFF3 are highly expressed in tissues containing mucus-producing cells. They play a key role in the maintenance of mucosal integrity and oncogenic transformation, growth, and metastatic extension of solid tumors [18–20]. TFF3 is expressed in goblet cells of the small and large intestines as well as in the intestinal metaplasia in the stomach [6–8].

Recent data indicate that serum TFFs, especially TFF3, could be potential biomarkers for the detection of gastric cancer. In a Japanese study conducted on 183 patients with gastric cancer and 280 healthy individuals, using a cutoff of 3.6 ng/mL for TFF3, the odds ratio for gastric cancer significantly increased (odds ratio 18.1; 95% confidence interval 11.2–29.2) and the sensitivity and specificity for predicting gastric cancer were 80.9 and 81.0%, respectively [11]. When comparing ROC curves of the pepsinogen I/II ratio, TFF3, and TFF3 plus pepsinogen I/II ratio, the TFF3 plus pepsinogen was found to have better results for gastric-screening marker than pepsinogen or TFF3 test only [11]. In another study conducted on 192 patients with gastric cancer and 1254 controls, the sensitivity and specificity of pepsinogen test for predicting gastric cancer were 67% and 82%, respectively, while a combination of serum TFF3 and pepsinogen test showed a sensitivity of 80 and specificity of 80% in detecting gastric cancer [21]. These previous results are consistent with the results from our study, on patients with EGC. We also compared the combination of serum TFF3 and pepsinogen with TFF3 or pepsinogen test only. The ROC curve of TFF3 for predicting EGC presence showed that the sensitivity, specificity, and area under the curve were 80.4%, 57.6%, and 0.729, respectively, using a cutoff of 6.73 ng/mL in the derivation cohort. The sensitivity of the combination of tests (87.5%) for EGC detection was superior to that of TFF3 (80.4%) or pepsinogen test alone (39.5%). Similarly, in the validation cohort, the ROC curve of TFF3 showed that the sensitivity, specificity, and area under the curve were 80.0%, 43.3%, and 0.651, respectively, using a cutoff of 6.73 ng/mL. The area under the curve for TFF3 plus pepsinogen I/II ratio (0.756) was higher than that for TFF3 alone (0.651) or pepsinogen I/II ratio alone (0.633). Additionally, the sensitivity of TFF3 plus pepsinogen (90.0%) was higher than that of TFF3 (80.0%) or pepsinogen test only (33.3%). TFF3 is a more useful marker than pepsinogen test for detection of EGC, and the combination of serum TFF3 plus pepsinogen is more effective than TFF3 or pepsinogen only.

We also evaluated the relationship between TFF3 and EGC histologic types according to differentiation and Lauren classification, respectively. We found that serum TFF3 levels in patients with differentiated-type gastric cancer were higher than in patients with undifferentiated-type histology, although these differences did not show statistical significance (*P* = 0.056). Serum TFF3 levels in patients with intestinal-type gastric cancer were significantly higher than in those with diffuse-type cancer (*P* = 0.028). Huang et al. [13] reported lower serum TFF3 concentrations in Chinese patients with differentiated-type and intestinal-type gastric cancers. Thus, the results of our study are not consistent with their report. In contrast, our study is highly consistent with the report of Kaise et al. [21] in Japan, which found that sensitivities of the TFF3 test alone and the combination of TFF3 and pepsinogen tests in diffuse-type adenocarcinoma were lower than those in intestinal-type cancer. Because TFF3 is strongly expressed by goblet cells in the epithelium of intestinal metaplasia of the stomach (according to the histopathogenesis of gastric cancer), a high TFF3 serum level would be expected in intestinal-type and differentiated-type gastric cancers. Further large studies are needed to explain these controversial results and discrepancies among previous studies.

EGD is an invasive examination used for early detection of gastric cancer, particularly in many asymptomatic subjects. Positive results of the combination of serum TFF3 and pepsinogen for gastric cancer could be helpful to encourage patients to undergo the EGD.

There were several limitations in this study. One was the relatively small sample size. However, our study showed similar results through two independent cohorts and this is the first study on the diagnostic usefulness of TFF3, which included only patients with EGC, and not AGC. Second, the proportion of diffuse-type EGC was small. However, the previous Korean study showed similar results and reported that diagnostic value of serum TFF3 for the diffuse-type cancer was somewhat decreased compared to that of intestinal-type gastric cancer although the proportion of EGC was 49.4% [17]. Third, control subjects in the derivation cohort were healthy Japanese and not Korean individuals. To overcome this and validate the present study, we analyzed a second independent Korean control cohort and results from both cohorts were similar. Fourth, our study did not show the detectability of precancerous lesions including atrophic gastritis by TFF3.

In summary, this study has shown that the serum TFF3 can be a more effective biomarker of EGC in Koreans than the pepsinogen test. Moreover, the combination of TFF3 and pepsinogen test had an increased diagnostic power as a screening modality. Additionally, results indicated the possibility of serum TFF3 level being associated with the histologic type and differentiation type in EGC. Further large studies are required to confirm the strong predictive power of serum TFF3 and the combination tests with TFF3 and pepsinogen in patients with AGC or EGC, as well as to clarify the role of serum TFF3 as a nonendoscopic biomarker in population-based screening for gastric cancer.

## Figures and Tables

**Table 1:** Baseline characteristics of patients with early gastric cancer and control groups in the derivation and validation cohorts.

Characteristics	Patients	Controls	*P* value
*Derivation cohort*			
*n*	281	708	
Sex, male, *n* (%)	213 (75.8)	272 (38.4)	<0.001
Age (years)	63.4 ± 9.3	67.4 ± 11.9	<0.001
TFF3 value (ng/mL)	9.37 ± 4.67	7.05 ± 3.28	<0.001
Male	9.21 ± 3.42	7.19 ± 3.86	<0.001
Female	9.87 ± 7.34	6.96 ± 2.86	0.002
*Helicobacter pylori* positivity (%)	136 (48.4)	NA	
Mean tumor size (mm)	22.0 ± 13.5	NA	
Submucosal invasion (%)	35 (12.5)	NA	
Lymphovascular invasion (%)	5 (1.8)	NA	
Histologic type (%)		NA	
Differentiated (WD, MD)	256 (91.1)		
Undifferentiated (PD, SRC)	25 (8.9)		
Lauren classification (%)		NA	
Intestinal	261 (92.9)		
Diffuse	20 (7.1)		
*Validation cohort*			
*n*	30	30	
Sex, male, *n* (%)	21 (70.0)	15 (50.0)	0.114
Age (years)	59.5 ± 10.7	66.6 ± 12.0	0.002
TFF3 value (ng/mL)	9.01 ± 4.21	6.92 ± 2.76	<0.001
Intestinal metaplasia, *n* (%)	13 (43.3)	3 (10.0)	0.004

Data are presented as the mean ± SD. TFF3: trefoil factor family 3; WD: well-differentiated adenocarcinoma; MD: moderately differentiated adenocarcinoma; PD: poorly differentiated adenocarcinoma; SRC: signet ring cell carcinoma; NA: not applicable.

**Table 2:** Evaluation of patients with early gastric cancer using pepsinogen and TFF3 levels.

	TFF3 (−)	TFF3 (+)	Total
Derivation cohort			
Pepsinogen test (−)	35 (20.6%)	135 (79.4%)	170
Pepsinogen test (+)	20 (18.0%)	91 (82.0%)	111
Total	55	226	281
Validation cohort			
Pepsinogen test (−)	3 (15.0%)	17 (85.0%)	20
Pepsinogen test (+)	3 (30.0%)	7 (70.0%)	10
Total	6	24	30

TFF3: trefoil factor family 3. Serum pepsinogen test (+): pepsinogen I < 70 ng/mL and pepsinogen I/II ratio < 3.
